# Lemierre's syndrome due to Klebsiella pneumoniae in a 63-year-old man with diabetes: a case report

**DOI:** 10.1186/1752-1947-6-97

**Published:** 2012-04-03

**Authors:** Musa A Garbati, Azeem M Ahsan, Ahmed M Hakawi

**Affiliations:** 1 Division of Infectious Diseases, Department of Medicine, King Fahad Medical City, 11525, Riyadh, Saudi Arabia

## Abstract

**Introduction:**

Lemierre's syndrome was originally documented to be caused by *Fusobacterium necrophorum*. It is a very rare condition with a prevalence of one to 14.4 instances per million. Its presentation is varied, not only in composition but also in the infecting organism. Treatment with anticoagulants has been controversial and applied only on a case-by-case basis.

**Case presentation:**

A 63-year-old Saudi man who had had uncontrolled diabetes mellitus for 47 years presented to our facility with a five-day history of swelling on the right side of his neck and fever. The swelling progressively increased in size and was associated with pain, dysphagia, odynophagia, change of voice ('hot potato voice'), and reduced appetite. Abscess content culture and sensitivity testing revealed *Klebsiella pneumoniae*. However, blood culture results were repeatedly negative. The abscess was incised and drained without any complication. Our patient was treated with clindamycin and cefuroxime. Warfarin was also administered concurrently for six weeks, for an isolated internal jugular vein thrombosis (IJV), with complete resolution of the thrombus. Normoglycemia was achieved and our patient was discharged after complete wound healing and the return of his biochemical parameters to normal.

**Conclusions:**

Only two cases of Lemierre's syndrome in patients with diabetes due to *K. pneumoniae *have been reported previously. A review of the literature suggested that an association exists between deep neck infections due to *K. pneumoniae *and diabetes mellitus. The reasons for this association are still not clear. This poses a question as to whether diabetes mellitus specifically predisposes these patients to infection with this organism. It is suggested that clinicians should consider infectious agents other than *F. necrophorum *in the causation of Lemierre's syndrome, especially in patients with diabetes.

## Introduction

Post-anginal sepsis, otherwise known as Lemierre's syndrome (LS) or necrobacillosis, was first reported by Courmont and Cade in 1900 [1]. The condition was better described by Andre Lemierre in 1936 [2] from a report of 20 cases. The hallmark of the syndrome, as was originally described by Lemierre, is a tetrad of acute oropharyngeal infection, septicemia, thrombophlebitis of the ipsilateral internal jugular vein (IJV), and secondary metastatic abscesses, most commonly to the lungs and joints. The syndrome has mainly been described in young, immunocompetent individuals, with a prevalence of one to 14.4 instances per million. This condition therefore requires a high index of suspicion for diagnosis [3]. The initiating infection is usually due to a Gram-negative anaerobe, *F. necrophorum *(previously known as *Bacillus fundiliformis*), but other organisms have also been implicated. Occasionally, no organisms were grown from clinical specimens [4].

LS was reported to be usually fatal prior to the discovery of antibiotics, with up to 90% mortality. The introduction of penicillin in the 1940s for oropharyngeal infections led to a significant drop in the incidence of the disease and its associated mortality (to as low as 5%) [5]. This has led to the disease becoming forgotten [6] and also to the appearance of incomplete forms of the syndrome.

We describe a rare case of LS in a patient with diabetes caused by *K. pneumoniae*. We also review the relevant literature.

## Case presentation

In November 2010, a 63-year-old Saudi man with background diagnoses of bronchial asthma and type 1 diabetes mellitus (DM I) of 47 years, presented to our facility with a five-day history of swelling on the right side of his neck and fever. The swelling progressively increased in size and was associated with pain, dysphagia, odynophagia, change of voice ('hot potato voice'), and reduced appetite. There was no prior history of shortness of breath, upper respiratory tract infection (URTI), dental problems or procedures. On examination our patient was found to be febrile (38.8°C), diaphoretic with a pulse rate of 115 beats per minute, a respiratory rate of 20 cycles per minute, BP of 119/69 mmHg and oxygen saturation of 99% at room air. There was a right submandibular swelling (5 × 5 cm) with all the cardinal features of acute inflammation (rubor, calor, tumor, dolor, and functio laesa). The first four of these signs were named by Celsus in ancient Rome (30-38 B.C.) and the last by Galen (A.D 130-200) [7].The right tonsil was enlarged and the uvula medially displaced. Examination of the respiratory system did not reveal any abnormalities.

Laboratory investigations revealed a white cell count of 10.5 × 10^9 ^cells/L (neutrophils 80.8%), hemoglobin 13.9 g/dL, platelet count 479 × 10^9 ^cells/L, erythrocyte sedimentation rate (ESR) 44 mm/hour and international normalized ratio (INR) of 3.1. Serum glucose was 23 mmol/L, total cholesterol 2.9 mmol/L, low-density lipoprotein (LDL) cholesterol 1.79 mmol/L, high-density lipoprotein (HDL) cholesterol 0.5 mmol/L and triglyceride 1.42 mmol/L. Hemoglobin A1c (HbA1c) was 9.9% on admission. Alanine aminotransferase (ALT) was 21 U/L, aspartate aminotransferase (AST) 6 U/L, uric acid 246 μmol/L, urea 5.5 mmol/L, creatinine 90 μmol/L, and total bilirubin 10.5 μmol/L. Abscess content culture and sensitivity testing yielded *K. pneumoniae*; however, blood culture results were negative. Serotyping of the *K. pneumonia *isolate from our patient was not performed.

**Figure 1:**
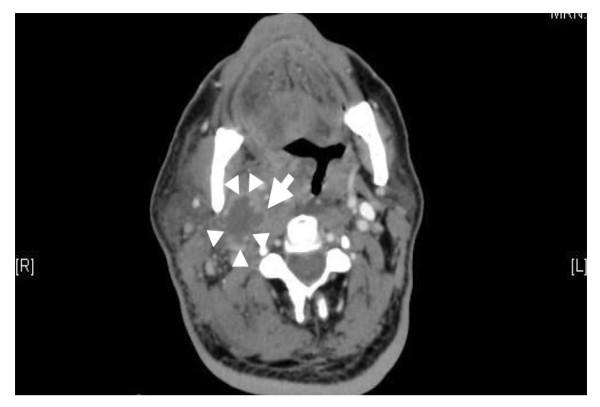
**Computed tomography (CT) scan of the neck region showing the abscess content (circle of arrowheads) and thrombosis in the right internal jugular vein (single arrow)**.

A computed tomography (CT) scan (Figure 1) of the neck region showed a multiloculated abscess (circle of arrowheads) with irregular peripheral enhancement in the right carotid space extending from the second to the fourth cervical vertebrae. The right sternomastoid muscle was swollen with surrounding inflammatory changes as well as multiple enlarged cervical lymph nodes. The fluid tracked through the pre-vertebral space from the C2 down to the C6 level, with a maximum anteroposterior (AP) diameter of 7 mm. It tracked into the right parapharyngeal space down to the supraglottic region. The palatine tonsils were enlarged, more significantly on the right side, and showed small areas of low attenuation. The right internal jugular vein (IJV) showed thrombosis and enhancement of its walls (single arrow in Figure 1) that extended intracranially into the right sigmoid and transverse venous sinuses. The thrombus also extended inferiorly down to the C7 level. Other features noted on the CT included a mass effect on the oropharynx and hypopharynx as well as the larynx. A CT scan of the chest was normal.

Our patient underwent incision and drainage of the abscess with no complications. He was treated with a combination of clindamycin and cefuroxime in addition to warfarin for six weeks. These interventions resulted in complete resolution of the thrombus. Normoglycemia was achieved before his discharge from hospital, and subsequent follow-up was uneventful. He was finally discharged from the clinic after complete wound healing and the return of his biochemical parameters to normal levels.

## Discussion

Historically, LS has been reported to most commonly affect young, healthy adults, although cases in children have also been reported [8]. Riordan recently reviewed 222 cases of LS and reported that in up to 89% the diagnosis was in individuals aged 10 to 35 years [9]. Since its initial description the condition has gone from being highly fatal, in the pre-antibiotic era, to a curable disease with the use of potent antimicrobials. The classic tetrad of features reported earlier in the century has given way to variants or incomplete forms of the disease with extension of the affected age group and variation in its microbiology. This has brought to the fore variants of or Lemierre-like syndromes, either due to isolation of organisms other than *F. necrophorum *[9] or presentations that were short of the tetrad originally described or due to the involvement of anatomic structures other than those reported earlier. A retrospective review of peri-tonsillar abscesses from Saudi Arabia between 2000 and 2004 by Al-Dakhail and Khan [10] revealed that out of the 65 samples sent for culture and sensitivity, 29.6%, 21%, and 3.7% yielded normal flora, group A Streptococci, and *Staphylococcus aureus*, respectively; while *K. pneumoniae *and *Streptococcus viridians *constituted 2.5% each of the isolates. No cases of *F. necrophorum *infection were reported in their patients. Some authors have questioned whether the isolation of *F. necrophorum *is really necessary for the diagnosis of LS [11]. Indeed a recent review reported 6% to 14% of cases of LS were associated with either negative cultures or organisms other than *F. necrophorum *[9]. It is of interest to note that the isolation of *F. necrophorum *requires strict anaerobic culture conditions. Other reasons that could have possibly led to negative blood cultures in our patient is prior antibiotic use or that *F. necrophorum *was overlooked, particularly by laboratory staff unfamiliar with its typical features and characteristics.

Involvement of *K. pneumoniae *in the causation of LS is rare, with only two previously reported cases [11,12] both of whom were also diabetic; therefore the question arose as to whether diabetes mellitus (DM) had a role in selecting this organism, or it was a mere coincidence? This association has been reported previously by Huang *et al. *[13], where 98.4% of their patients with diabetes had infections due to *K. pneumoniae*. A recent review from Singapore [14] by Lee and Kanagalingam documented about 50% of their patients with diabetes with deep neck abscesses had *K. pneumoniae *isolated from the collections. Some of these abscesses were found to be caused by the hypermucoviscosity (HV) phenotype of *K. pneumoniae*, especially K1 and K2 isolates which tend to be significantly more resistant to phagocytosis than non-K1/K2 isolates [15].

Like other Gram-negative bacteria, *F. necrophorum *produces lipopolysaccharide, which behaves like a classical endotoxin with virulence in many animal models [9]. Development of the disease has been described to occur in three stages: (1) infection of deep pharyngeal tissue; (2) invasion of the lateral pharyngeal space, resulting in IJV thrombophlebitis; and (3) embolic spread of the infection. The exact etiology and pathogenesis of LS is still unknown, but lymphatic or direct spread of infection from the thrombophlebitis of the tonsillar veins causing thrombosis of the IJV due to direct involvement of the alveolar tissue of the neck has been suggested as a possible mechanism [16].

In the absence of controlled trials to determine the optimum therapy for LS, most authors based their choice of antimicrobial agent(s) mainly on personal experience and anecdotal evidence. Metronidazole is usually the most preferred agent though some authors used either a carbapenem or a penicillin/β-lactamase inhibitor combination for a total of six weeks, but the optimal duration of treatment is not known.

The use of anticoagulants has been controversial, as no controlled trials exist. Some authors observed persistence of fever, worsening clinical condition and ongoing radiographic evidence of pulmonary emboli until full anticoagulant therapy has been initiated [17]. Routine anticoagulation is not generally required for all cases of IJV thrombosis in the absence of convincing data from randomized studies. However, anticoagulation has been frequently advocated in situations where untoward consequences are expected such as the involvement of the sigmoid or cavernous sinuses. Another indication for anticoagulation in patients with LS would be suppurating thrombophlebitis of the sigmoid sinus propagating to the cavernous sinus.

## Conclusions

Cases of LS are rare. It may be caused by organisms not originally described by Andre Lemierre. Clinicians should therefore look out for organisms other than *F. necrophorum *when clinical suspicion is high and also broaden the antimicrobial cover. In all cases reported due to *K. pneumoniae*, the patients happen to be diabetic; therefore the question arose as to whether diabetes mellitus specifically selects for this organism. Available evidence suggests this could be the case. The role of anticoagulation has been controversial and since no randomized trials are available, this approach has only been applied on a case-by-case basis. Accurate and timely diagnosis is critical because untreated disease is usually fatal. Primary care providers should therefore be aware of the syndrome in patients with oropharyngeal infection who subsequently show signs of systemic illness or pulmonary involvement.

## Consent

Written informed consent was obtained from the patient for publication of this case report and any accompanying images. A copy of the written consent is available for review by the Editor-in-Chief of this journal.

## Competing interests

The authors declare that they have no competing interests.

## Authors' contributions

MAG designed and prepared the manuscript. AMA managed our patient and also reviewed the manuscript. AMH reviewed the manuscript. All authors have read and approved the final manuscript for publication.
